# The genetic and epigenetic profile of serum S100β in the Lothian Birth Cohort 1936 and its relationship to Alzheimer’s disease

**DOI:** 10.12688/wellcomeopenres.17322.2

**Published:** 2022-01-27

**Authors:** Danni A Gadd, Robert I McGeachan, Robert F Hillary, Daniel L McCartney, Sarah E Harris, Roy A Sherwood, N Joan Abbott, Simon R Cox, Riccardo E Marioni

**Affiliations:** 1 Institute of Genetics and Cancer, University of Edinburgh, Edinburgh, Other (Non-U.S.), EH4 2XU, UK; 2 Centre for Discovery Brain Sciences, University of Edinburgh, Edinburgh, Other (Non-U.S.), EH8 9JZ, UK; 3 Lothian Birth Cohorts, University of Edinburgh, Edinburgh, Other (Non-U.S.), EH8 9JZ, UK; 4 Department of Psychology, University of Edinburgh, Edinburgh, Other (Non-U.S.), EH8 9JZ, UK; 5 Department of Clinical Biochemistry, King's College Hospital NHS Foundation Trust, London, Other (Non-U.S.), SE5 9RS, UK; 6 Institute of Pharmaceutical Science, King's College London, London, Other (Non-U.S.), WC2R 2LS, UK

**Keywords:** Epigenetic, Genetic, S100β, Inflammation, EWAS, GWAS, Alzheimer's disease

## Abstract

**Background:** Circulating S100 calcium-binding protein (S100β) is a marker of brain inflammation that has been associated with a range of neurological conditions. To provide insight into the molecular regulation of S100β and its potential causal associations with Alzheimer’s disease, we carried out genome- and epigenome-wide association studies (GWAS/EWAS) of serum S100β levels in older adults and performed Mendelian randomisation with Alzheimer’s disease.

**Methods:** GWAS (N=769, mean age 72.5 years, sd = 0.7) and EWAS (N=722, mean age 72.5 years, sd = 0.7) of S100β levels were performed in participants from the Lothian Birth Cohort 1936. Conditional and joint analysis (COJO) was used to identify independent loci. Expression quantitative trait locus (eQTL) analyses were performed for lead loci that had genome-wide significant associations with S100β. Bidirectional, two-sample Mendelian randomisation was used to test for causal associations between S100β and Alzheimer’s disease. Colocalisation between S100β and Alzheimer’s disease GWAS loci was also examined.

**Results:** We identified 154 SNPs from chromosome 21 that associated (P<5x10 ^-8^) with S100β protein levels. The lead variant was located in the *S100β* gene (rs8128872, P=5.0x10 ^-17^). We found evidence that two independent causal variants existed for both transcription of *S100β* and S100β protein levels in our eQTL analyses *. *No CpG sites were associated with S100β levels at the epigenome-wide significant level (P<3.6x10 ^-8^); the lead probe was cg06833709 (P=5.8x10 ^-6^), which mapped to the *LGI1* gene. There was no evidence of a causal association between S100β levels and Alzheimer’s disease or vice versa and no evidence for colocalisation between *S100β *and Alzheimer’s disease loci.

**Conclusions:** These data provide insight into the molecular regulators of S100β levels. This context may aid in understanding the role of S100β in brain inflammation and neurological disease.

## Introduction

The calcium-binding protein S100 beta (S100β) has been suggested as a biomarker for central nervous system disease ^1^. Expressed most commonly in astrocytes, its cytoplasmic location and calcium-binding capability allows S100β to mediate calcium homeostasis, cell proliferation and survival intracellularly, while also triggering the RAGE-associated inflammatory response when secreted extracellularly ^2^. Part of pro-inflammatory danger-associated molecular patterns (DAMPs), elevated S100β is linked to cytokine cascades in the brain ^3, 4^.

Although the exact pathophysiology is still unknown, a number of small-scale studies have reported elevated circulating or cerebrospinal fluid (CSF) S100β levels in individuals with nervous system injury, neuroinflammatory conditions, white matter ageing and Alzheimer’s dementia and delirium ^4– 9^. Following traumatic brain injury, elevated S100β plasma levels have been shown to precede increases in intracranial pressure ^1^. Blood-based levels of S100β have also been found to be elevated in small vessel disease and associated with cognitive decline ^10, 11^. Activated astrocytes expressing high levels of S100β have been profiled at plaques in the hippocampus, temporal lobe, frontal lobe and pons in individuals with Alzheimer’s disease ^12^. The *S100β* gene was also identified as a site of differential DNA methylation (DNAm) relating to Braak staging in a previous epigenome-wide association study (EWAS) of cortical post-mortem tissues (n=159) ^13^. 

Whether S100β has a direct involvement in the pathogenesis of Alzheimer’s disease is still unclear. Further to its potential role in inflammatory exacerbation in the brain, research suggests that at nanomolar concentrations, S100β can have protective and neurotrophic effects ^14^. Despite the widely discussed biological importance of S100β, the possible epigenetic regulators of the protein have not been investigated. One study using blood spots taken at birth has previously identified two genetic associations in relation to circulating S100β levels; rs62224256 on chromosome 21, 21kb downstream of the pericentrin gene ( *PCNT*) and rs28397289 on chromosome 6, within the human leukocyte antigen ( *HLA*) region ^15^. Elucidating the mechanisms that determine inter-individual variation in circulating S100β levels, in healthy individuals, may therefore provide insight into S100β’s role in health and disease. Further genetic mapping of S100β may also facilitate causal association tests with disease endpoints.

Here, we perform genome- and epigenome-wide association studies (GWAS/EWAS) of S100β in relatively healthy older adults from the Lothian Birth Cohort 1936 (LBC1936). We then use genetic instruments identified for S100β to test for bidirectional causal associations with Alzheimer’s disease, via two-sample Mendelian randomisation. Demographic information for the GWAS (N=769) and EWAS (N=722) sample groups are summarised in Table 1.

**Table 1.** Demographics summary for the EWAS and GWAS sample populations.

Phenotype	EWAS		GWAS	
	n (%)	Mean (sd)	n (%)	Mean (sd)
Maximum sample (n)	722		769	
S100β (μg/L)		0.086 (0.035)		0.085 (0.034)
Sex (Female)	340 (47.1)		367 (47.7)	
Age at S100β (years)		72.5 (0.7)		72.5 (0.7)
Body Mass Index (kg/m ^2^)		27.9 (4.3)		27.9 (4.3)
EpiSmokEr		0.84 (5.2)		

## Methods

### The Lothian Birth Cohort 1936

The Lothian Birth Cohort 1936 (LBC1936) is a longitudinal study of cognitive ageing. Cohort members were born in 1936 and took part in the Scottish Mental Survey 1947 at age 11 years. Approximately 60 years later, those individuals that were living mostly within the Edinburgh area were re-contacted (n = 1,091, recruited at mean age 70 years). Recruitment and testing of the LBC1936 cohort have been described previously ^16, 17^. Briefly, the data collection has included detailed phenotypic, biological and cognitive sampling, over a series of follow-up waves (approximately every 3–4 years since recruitment). Body mass index (BMI) was measured at clinic visits and recorded in kg/m ^2^. The S100β data available in this study were from the second wave of testing, at around three years after the Wave 1 visit to the clinic. DNA methylation data were also collected at the same time-point as S100β sampling. Genotyping was performed on DNA from blood samples collected at Wave 1.

### S100β measurement

Serum samples were obtained from participants during the main physical and cognitive testing appointment at Wave 2. After collection, samples were stored at −80 °C at the Wellcome Trust Clinical Research Facility, Western General Hospital, Edinburgh, until the conclusion of the wave. They were then transferred to the Department of Clinical Biochemistry, King's College London using cold-chain logistics, where they were stored at −20 °C until assays were conducted using a chemiluminescence immunoassay S100β kit (catalogue number 314701, distributed by DiaSorin, Berks, UK) on a LIAISON chemiluminescence analyzer. The lag between sample dispatch at the end of sampling and assay completion (i.e., time stored at −20 °C rather than −80 °C) was an average of 44 days (SD = 26) for four batches. The minimal detectable concentration of the assay was 0.02 μg/L.

### Genotyping

LBC1936 DNA samples were genotyped at the Edinburgh Clinical Research Facility using the Illumina 610-Quadv1 array (Wave 1; n = 1005; mean age: 69.6 ± 0.8 years; San Diego). Preparation and quality control steps have been reported previously ^18^. SNPs were imputed to the 1000 G reference panel (phase 1, version 3). Briefly, individuals were excluded on the basis of sex mismatches, relatedness, SNP call rate of less than 0.95, and evidence of non-European ancestry. SNPs with a call rate of greater than 0.98, minor allele frequency in excess of 0.01, and Hardy-Weinberg equilibrium test with P ≥ 0.001 were included in analyses. Only SNPs with a minor allele frequency > 0.05 and imputation quality > 0.6 were retained. The remaining 8,489,963 SNPs were filtered to remove those that had a minor allele count < 25. A total of 7,307,523 SNPs were available for GWAS analyses.

### DNA methylation

DNA methylation from whole blood at Wave 2 of the Lothian Birth Cohort 1936 was measured using the Illumina 450 K methylation array at the Edinburgh Clinical Research Facility. Complete details of quality control steps taken to process the dataset have previously been described ^19^. Briefly, raw intensity data were background-corrected and normalised using internal controls. Manual inspection facilitated the removal of low quality samples presenting issues relating to bisulphite conversion, staining signal, inadequate hybridisation or nucleotide extension. Further quality control analyses were performed to exclude probes with low detection rate <95% at *P* < 0.01 and samples with a low call rate (<450,000 probes detected at *p*-values of less than 0.01) were also excluded. Finally, samples were removed if there was a poor match between genotype and incorrect DNA methylation-predicted sex, or SNP control probes. DNA methylation at Wave 2 was processed in three sets (n=256, 461 and 5, for sets 1, 2, and 3, respectively). In total, there were 459,309 CpG sites used in EWAS analyses. Lothian Birth Cohort Wave 2 DNA methylation data were used to generate an epigenetic score for smoking – known as EpiSmokEr ^20^ in the sample. This score utilises previously derived weights calculated in an independent sample and has been previously shown to robustly reflect smoking status ^20^.

### S100β sample preparation

**Figure 1.**
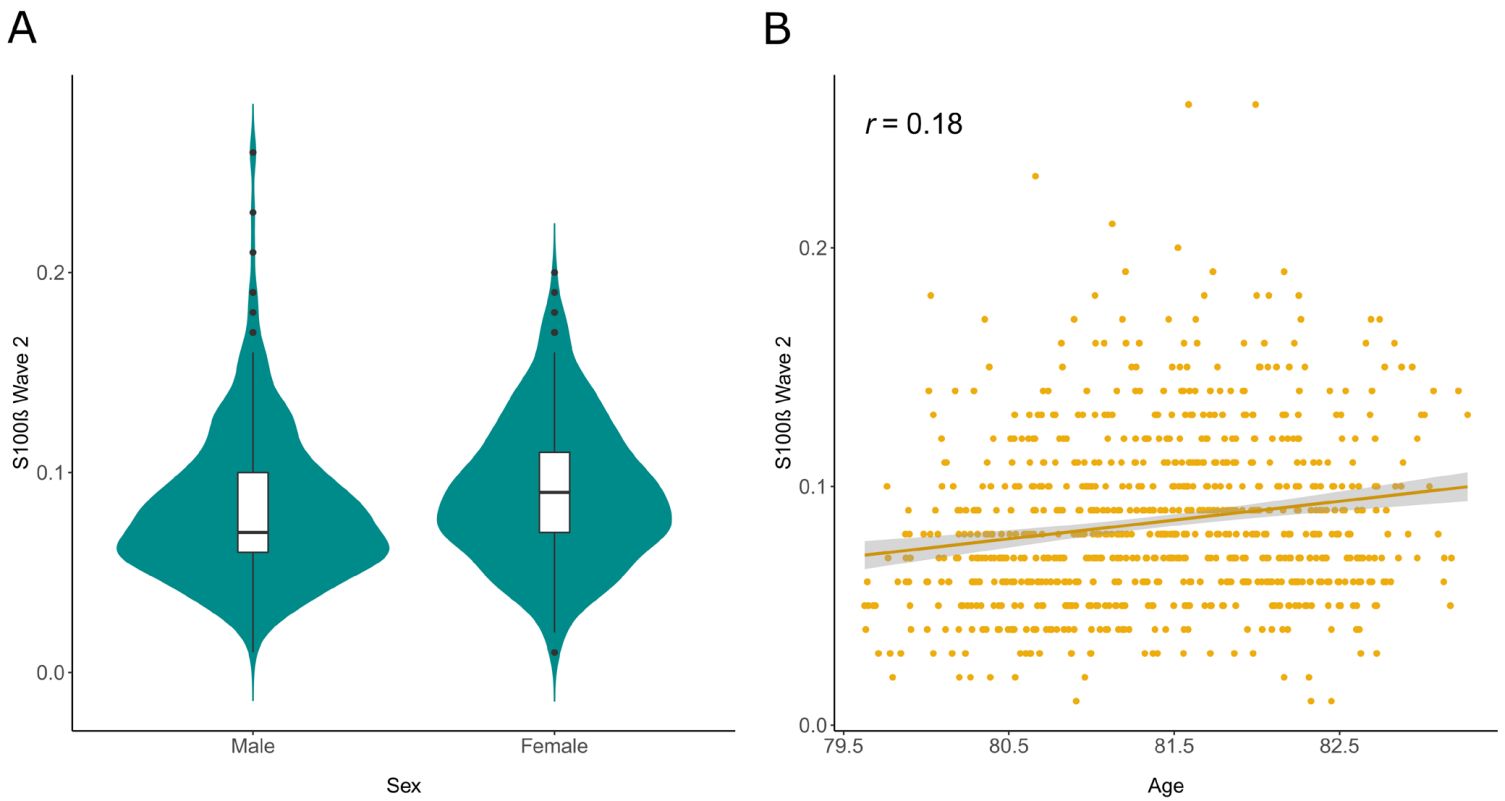
S100β in Wave 2 of the Lothian Birth Cohort 1936 (N=722). **A,** Violin plot to illustrate differences in S100β by sex. **B,** Scatterplot with regression line and 95% CIs for S100β by age at serum sampling with a Pearson’s correlation coefficient ( *r*) annotated. S100β is plotted in μg/L units in all instances and age is given in years.

There were 834 individuals with S100β concentrations recorded at Wave 2 of the Lothian Birth Cohort study. Six measurements greater than four standard deviations from the mean were excluded, as per previous analyses that utilised this sample ^9^. There were 769 individuals with genome-wide genetic data (mean 72.5 years, sd = 0.7) and 722 individuals with epigenome-wide DNA methylation data (mean 72.5 years, sd = 0.7) available. Table 1 summarises demographic information for these sample populations. In the maximum sample available in GWAS and EWAS (N=722), serum S100β levels were higher in females (beta = 0.26, SE = 0.07, P = 2.2×10 ^-4^) and older individuals (correlation of 0.18 and beta = 0.16 per year, SE = 0.03, P = 2.9×10 ^-6^ in linear models) ( Figure 1).

Body mass index (BMI) and smoking are common lifestyle covariates that have well-documented DNAm signatures ^21, 22^; we therefore tested whether these traits should be adjusted for in our analyses. S100β levels were positively associated with body mass index (BMI) (beta = 0.10 per kg/m ^2^, SE = 0.03, P = 2×10 ^-3^), but did not associate with the DNAm-based score for smoking, EpiSmokEr (beta = -0.01 per unit increase in the DNAm smoking score, SE = 0.04, P = 0.78). For this reason, BMI was included as a covariate in all analyses.

S100β protein levels were transformed by rank-based inverse normalisation and regressed onto age, sex, BMI at Wave 2 (kg/m ^2^) and four genetic principal components of ancestry in separate analyses groups (EWAS N=722, GWAS N=769). Standardised residuals (mean = 0, variance = 1) from these linear regression models were brought forward as the protein level variable for the respective analyses.

Data quality control and preparation was conducted in R (Version 4.0.3) ^23^

### Genome-wide association study (GWAS)

Linear regression was used to assess the effect of each of the 7,307,523 available SNPs on the levels of S100β via PLINK (Version 1.9) ^24^. Genome-wide stepwise conditional analysis was performed through GCTA-COJO using the ‘cojo-slct’ option to identify independent variants. Individual level genotype data were used for the reference linkage disequilibrium (LD) structure along with default settings of the software ^25^. The variance (r ^2^) in S100β levels that could be explained by this variant was calculated as follows: r ^2^ = 2 × MAF × (1-MAF) × beta ^2^, where beta = effect size of the SNP and MAF = the effect allele frequency.

### Expression quantitative trait loci (eQTL) colocalisation

We cross-referenced sentinel *cis* pQTLs that were selected by GCTA-COJO analyses with publicly available *cis* eQTL data taken from the eQTLGen consortium ^26^. The cis eQTLs were subset to the same chromosome as the cis pQTL. A 200 kb region (either upstream or downstream) was extracted from our GWAS summary statistics for S100β to capture *cis* effects within 100 kb of the target gene ^27^. eQTLs for this region were then extracted from the eQTLGen consortium summary statistics for the *S100β* region. The shared SNPs across transcripts for *S100β* and S100β protein levels were then tested for colocalisation using the coloc package ^28^ (Version 5.1.0) in R, with five hypotheses in Bayesian tests with default priors ^28^. In addition to the null hypothesis (no causal variant), hypothesis 1 indicated a causal variant was present for S100β protein levels only. Hypothesis 2 indicated that there was a causal variant for the *S100β* transcript only. Hypothesis 3 indicated that there were independent causal variants for both *S100β* transcription and S100β protein levels. Hypothesis 4 indicated that there were two association signals that contributed to both *S100β* gene expression and S100β protein levels. A posterior inclusion probability (PP) > 0.95 was taken as the threshold for hypothesis testing.

### Mendelian randomisation

Two-sample, bidirectional Mendelian Randomisation (MR) was used to test for potentially causal associations between S100β protein levels and Alzheimer’s disease. Associations from separate GWAS were used as genetic instruments. As allele assignment is randomised, the SNPs associated with the exposure are randomised to the effects of confounders and likely to be causally upstream of the exposure ^29^. Summary statistics from a GWAS performed by Jansen *et al.* ^30^ were used as the Alzheimer’s disease dataset (13,367,299 SNPs, with N=71,880 cases, and N=383,378 controls). The GWAS summary statistics for S100β were sourced from the analyses in this study that used samples from the Lothian Birth Cohort 1936 (N=769). Importantly, the Alzheimer’s disease summary statistics were based on a meta-analysis of cohorts that were independent of the Lothian Birth Cohort 1936. All analyses were performed using the TwoSampleMR package (Version 0.5.6) in R ^29^. One assessment quantified the association between S100β levels as the exposure and Alzheimer’s disease as the outcome. A second assessment then quantified the association between Alzheimer’s disease as the exposure and S100β levels as the outcome. In each of the MR analyses, clumping was used to prune SNPs for linkage disequilibrium (LD) at r ^2^ < 0.001. When testing the association with S100β as the exposure and Alzheimer’s disease as the outcome, only one of the 154 SNPs with P<5×10 ^-8^ (rs8128872; the sentinel variant identified by GCTA-COJO analyses in our GWAS of S100β) remained after LD pruning. The effect estimate for S100β to Alzheimer’s disease was therefore determined using the Wald ratio test (a ratio of effect per risk allele on trait to effect per risk allele on protein levels). An F statistic for the strength of the association between the sentinel SNP and the exposure was calculated using the method: F = ((N-k-1) / k) × (r ^2^ / (1-r ^2^)), where N = sample size, k = number of SNPs and r ^2^ = variance explained in S100β levels by the genetic instruments. The r ^2^ statistic was calculated as follows: r ^2^ = 2 × MAF × (1-MAF) × beta ^2^, where beta = effect size of the SNP and MAF = the effect allele frequency. When testing causal associations with Alzheimer’s disease as the exposure and S100β as the outcome, 30 of the 2,357 SNPs with P<5×10 ^-8^ remained after LD pruning and 29 were present in the S100β summary statistics. Multi-method MR was then performed using the 29 SNPs from the Jansen *et al.* ^30^ summary statistics. As multiple independent variants were identified, a multi-method MR approach was chosen ^29^. Unity between the estimates from these methods indicates that the results are more likely to be robust. The MR Egger approach did not find strong evidence of horizontal pleiotropy present (non-significant MR-Egger intercept).

### Colocalisation 

Colocalisation analysis can be used to derive the probability that common genetic variants are shared between two phenotypes in a given region of the genome. The coloc package (Version 5.1.0) was used to conduct colocalisation analyses for the sentinel SNP in the S100β region and the Jansen *et al.* ^30^ summary statistics for Alzheimer’s disease GWAS. Each dataset was subset to a 200 kb section (upstream or downstream) surrounding the sentinel SNP on chromosome 21. Rare variants with MAF < 0.01 and variants with missing MAF were excluded from the analysis. A total of 1,346 variants were included for Alzheimer’s disease and 1,010 variants were included for S100β. A single causal variant assumption is made in the analysis that there is one causal variant per trait and the probability of colocalisation between loci can be derived. Four hypotheses were used in Bayesian tests with default priors ^28^, as per the eQTL colocalisation tests, but for the presence of Alzheimer’s disease causal variants in the same region as *S100β*. In addition to the null hypothesis (no causal variants in the region assessed), hypothesis 1 indicated a causal variant was present for Alzheimer’s disease only. Hypothesis 2 indicated that there was a causal variant for S100β levels only. Hypothesis 3 indicated that there were independent causal variants for both Alzheimer’s disease and S100β levels. Hypothesis 4 indicated that a common variant contributed to both Alzheimer’s disease and S100β levels. A posterior inclusion probability (PP) > 0.95 was taken as the threshold for hypothesis testing.

### Epigenome-wide association study (EWAS)

DNA methylation data were regressed onto age, sex, DNAm set, DNAm batch, BMI at Wave 2 (kg/m ^2^), the DNAm-based smoking score EpiSmokEr ^20^, four genetic principal components and the measured levels of five immune cells (eosinophils, basophils, lymphocytes, neutrophils and monocytes). EWAS was conducted using OmicS-data-based complex trait analysis (OSCA) ^31^. The MOMENT method was used to test for associations between S100β levels and DNAm at individual CpG sites. MOMENT is a mixed-linear-model-based method that is able to account for unobserved confounders and the correlation between distal probes that may be introduced by these confounders. CpG sites were the independent variables and the dependent variable was the S100β protein residuals.

## Results

### Genetic profiling of S100β

**Figure 2.**
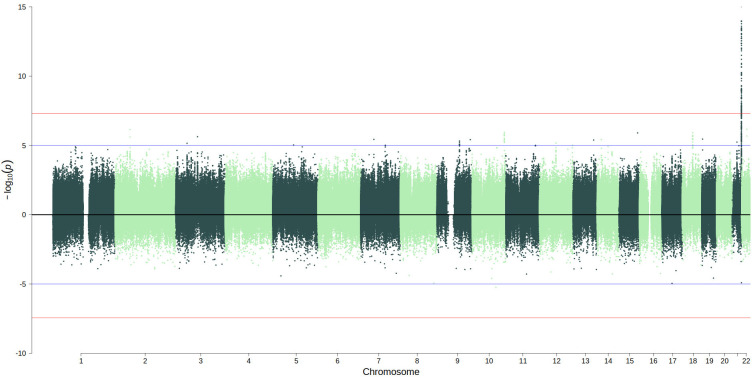
Miami plot of the GWAS (upper panel; N=769) and EWAS (lower panel, N=722) of S100β. Blue lines indicate a suggestive threshold of P<1×10 ^-5^; red lines indicate genome-wide thresholds of P<5×10 ^-8^ (GWAS) and P<3.6×10 ^-8^ (EWAS).

**Figure 3.**
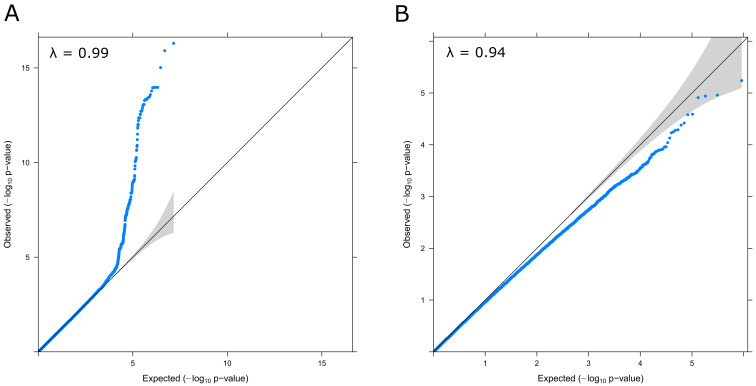
QQ Plots and Genomic Inflation statistics for the GWAS ( **A**) and EWAS ( **B**) of S100β in the LBC1936 sample. Lambda (λ) values are annotated in each case.

The linear regression genome-wide association study identified 154 SNPs ( Figure 2, full summary statistics are available in the Extended Data) on chromosome 21 that were associated with S100β levels at P<5×10 ^-8^ (N=769). There was no evidence of genomic inflation (lambda = 0.99; Figure 3). Conditional and joint analysis (GCTA-COJO) resulted in the identification of one independent pQTL rs8128872 (COJO beta = -0.46, SE = 0.05, P = 3.2×10 ^-18^) associated with S100β levels. The rs8128872 variant was found to explain 9% of the variance in S100β levels. The pQTLwas a *cis* variant (located 1,419,889 base pairs downstream of the transcription start site of the *S100β* gene on chromosome 21). There was strong evidence (posterior probability (PP) > 0.95) that two independent causal variants existed for both transcription of *S100β* and S100β protein levels in our eQTL analyses for the *S100β* locus (Hypothesis 3: Posterior Probability (PP) = 1.0, Hypotheses 1, 2 and 4 = 0).

Two-sample Mendelian randomisation (MR) was used to test for a causal association between S100β serum levels (using our *cis* GWAS data) and Alzheimer’s disease. As only one significant SNP remained after LD pruning (F = 76.67, indicating a strong effect of the instrument on the S100β exposure), the causal effect estimate was determined using the Wald ratio. There was no evidence of an effect of S100β serum levels on risk of Alzheimer’s disease (P = 0.95); ( Table 2). Similarly, there was no evidence to suggest that a causal relationship was present between Alzheimer’s disease and serum S100β (P > 0.05). Colocalisation analyses provided further evidence that the loci for Alzheimer’s disease and S100β were not localised together (Hypothesis 2: Posterior Probability (PP) = 0.99, Hypothesis 3: PP = 0.01, Hypotheses 1 and 4 = 0).

**Table 2.** Mendelian Randomization summary statistics for two-sample, bidirectional tests between S100β serum levels and Alzheimer's disease.

Exposure	Outcome	Method	N SNP	Odds Ratio	Beta	SE	P
S100β	Alzheimer's disease	Wald ratio	1	1.0002	3×10 ^-4^	4.9×10 ^-3^	0.95
Alzheimer's disease	S100β	MR Egger	29	N/A	0.33	0.51	0.52
Alzheimer's disease	S100β	Weighted median	29	N/A	-0.21	0.53	0.69
Alzheimer's disease	S100β	Inverse variance weighted	29	N/A	-0.53	0.38	0.16
Alzheimer's disease	S100β	Simple mode	29	N/A	-1.02	0.98	0.31
Alzheimer's disease	S100β	Weighted mode	29	N/A	-0.03	0.49	0.96

### Epigenetic profiling of S100β

No CpGs were significantly associated (P<3.6×10 ^-8^) with S100β levels in the EWAS study (N=722) ( Figure 2, full summary statistics are available in the Extended Data). The site with the lowest p-value (P = 5.8×10 ^-6^) was cg06833709, which is located within the *LGI1* region, known to encode the leucine-rich glioma inactivated 1 protein (known as epitempin). There was no evidence of genomic inflation (lambda = 0.94, Figure 3).

## Discussion

We have characterised the genetic and epigenetic profiles of S100β, a circulating protein that has been associated with brain inflammation and neurological disease pathology. We identified a genome-wide significant *cis*-pQTL (rs8128872, P = 5.0×10 ^-17^) that was associated with inter-individual variability in circulating S100β levels and found evidence that this pQTL was likely to be distinct from the eQTL for *S100β* transcription. Mendelian randomisation suggested no evidence of a causal association between S100β and Alzheimer’s disease or vice versa. Furthermore, there were no CpG probes that had epigenome-wide significant associations with S100β.

Whereas the study size was modest for our GWAS and EWAS, previous investigations of similar sample sizes have identified genome-wide SNP and epigenome-wide CpG correlates of protein levels ^32– 35^. These include proteomic analyses in the age homogeneous Lothian Birth Cohort 1936 sample that we use in our study ^34, 35^. Despite this, larger GWAS and EWAS efforts may help to identify additional loci, which could be used for genetic correlation and more detailed Mendelian randomisation analyses.

Our identification of a single sentinel SNP for S100β suggests that though limited, there is some evidence for genetic regulation of this protein in blood. The *cis*-pQTL that we identify (rs8128872) adds to the genetic profile of S100β generated in a previous GWAS analysis, which identified two SNPs in the *PCNT* and *HLA* regions ^15^. While our EWAS suggested that there is no epigenome-wide signature of differential DNAm relating to circulating S100β levels, the CpGs with the lowest P values in associations were situated within loci such as *LGI1*. The leucine-rich glioma inactivated 1 protein is encoded by *LGI1*, primarily locates at neuronal synapses and dysregulation of this protein due to LGI1 autoimmunity has been directly associated with limbic encephalitis ^36, 37^. Whether methylation at this CpG is associated with S100β should therefore be confirmed by replication.

The lack of evidence for a causal relationship between S100β and Alzheimer’s disease suggests either that 1) S100β levels in the blood are not directly related to the disease, or 2) any associations are more modest than this study is powered to reliably detect. The GWAS sample size was relatively small and it is imperative that our GWAS and Mendelian randomisation results are independently validated by future cohorts that have S100β measurements available. This will further elucidate the likelihood of a causal relationship between S100β and Alzheimer’s disease. Studies implicating S100β as a candidate marker for dementia are often performed in cerebrospinal fluid ^5, 7, 8^, which may provide a closer reflection of brain pathology and comparisons between blood and CSF S100β levels may therefore yield differing conclusions. However, in the Lothian Birth Cohort 1936 sample we use in this study, serum S100β levels have cross-sectionally been associated with poorer general fractional anisotropy ^9^, a marker of brain ageing that is associated with increased risk of cognitive decline and dementia ^38^. Given its role as a mediator in inflammatory cascades within the brain ^2, 4^, it is likely that S100β serum levels may be modulated by multiple factors that could be independent of Alzheimer’s disease, or indirect from the dementia-associated pathology occurring in the brain. This may be evidenced by our lack of causal association to Alzheimer’s disease and these pathways should be explored to delineate targets for therapeutic interventions that may alter neuroinflammation through S100β mediation.

There are several limitations to our study. First, the Lothian Birth Cohort 1936 are of European ancestries, have little variation in age and selection biases may exist in this cohort, who are considered to be of higher socioeconomic class to the wider Scottish population ^16^. Therefore, these findings may not generalise to individuals of different ethnic backgrounds, age profiles or socioeconomic groups, though this also means that we are not largely reliant on statistical adjustment for these confounders. Second, our data are from relatively healthy individuals, none of whom reported a diagnosis of dementia at recruitment. It is plausible that analyses in individuals in specific diagnoses groups may yield differing findings. Finally, while the amount of information regarding brain pathology and neurological disease biology is limited when using blood measurements, many blood-based biomarkers have been found to predict and offer insight into Alzheimer’s disease ^39^. Therefore, approaches that seek to triangulate between blood, cerebrospinal fluid and brain tissues may strengthen the identification of biomarker signals in future.

## Conclusion

We have established evidence for modest genetic, but not epigenetic contributions to the levels of S100β, a protein marker for brain inflammation and neurological disease. We found no evidence for a causal relationship between serum S100β and Alzheimer’s disease. Future studies should seek to corroborate these findings across blood, cerebrospinal fluid and brain tissue.

## Data availability

### Underlying data

Lothian Birth Cohort 1936 data are not publicly available due to them containing information that could compromise participant consent and confidentiality. Lothian Birth Cohort 1936 data are available on request from the Lothian Birth Cohort Study, University of Edinburgh.

If you are interested in working with the Lothian Birth Cohort 1936 data, you must complete a Data Request Form, indicating the variables you wish to access from the Data Dictionaries. Data Dictionaries and Data Request Forms are freely accessible at the following website: https://www.ed.ac.uk/lothian-birth-cohorts/data-access-collaboration. Completed forms must then be sent to Dr Simon Cox ( simon.cox@ed.ac.uk) for approval.

All code is available at the following Gitlab repository: https://github.com/DanniGadd/GWAS-and-EWAS-of-S100-.

### Extended data

Zenodo: The genetic and epigenetic profile of serum S100β in the Lothian Birth Cohort 1936 and its relationship to Alzheimer's disease, https://doi.org/10.5281/zenodo.5591776.

This project contains the following extended data files:

- s100b_EWAS_output.mlma (EWAS summary statistics for the epigenetic association study of S100β levels, for each of the 459,309 CpG probes tested)
- s100b_GWAS_output.txt (GWAS summary statistics for the genetic association study of S100β levels, including the 154 variants that had P < 5×10 ^-8^)

### Reporting guidelines

Zenodo: The STROBE reporting checklist for observational studies, https://doi.org/10.5281/zenodo.5591776

Data are available under the terms of the Creative Commons Zero "No rights reserved" data waiver (CC0 1.0 Public domain dedication).

### Ethical approval and consent

Ethical approval for LBC1936 was obtained from the Multi-Centre Research Ethics Committee for Scotland (MREC/01/0/56) and the Lothian Research Ethics committee (LREC/1998/4/183; LREC/2003/2/29). All participants provided written informed consent and the study was performed in accordance with the Helsinki declaration.
