# Supplementary material for: The genetic and epigenetic profile of serum S100β in the Lothian Birth Cohort 1936 and its relationship to Alzheimer’s disease
Source: Wellcome Open Res. 2022 Jan 27;6:306. Originally published 2021 Nov 10. [Version 2] doi: 10.12688/wellcomeopenres.17322.2 (PMC8686327; doi:10.12688/wellcomeopenres.17322.2)
Supplement: Peer review history

# Peer review: Reviewer response for version 1

**DOI:** 10.21956/wellcomeopenres.19151.r47163

**Published:** 2021-12-17

**Authors:** Manu Sharma

**Affiliations:** 1 Centre for Genetic Epidemiology, Institute for Clinical Epidemiology and Applied Biometry, University of Tübingen, Tübingen, Germany

The identification of relevant biomarker signatures for age-related neurodegenerative diseases is an area that merits well-designed studies. The study by Gadd *et al*. is such an effort in which the authors have performed genome and epigenome-wide association studies (GWAS/EWAS) of serum S100β levels in older adults. Subsequently, they also performed Mendelian randomization (MR) with Alzheimer’s disease (AD) to infer a causal relationship between S100β levels and AD.

 It is a well-designed study. The methodology applied and subsequent interpretation of their findings are well interpreted. I do not have any major comments regarding their study design and interpretation.

 Minor comments:

 The authors should explain in detail their MR design and analysis. The study cohort is comparatively small. The authors should explain this as a caveat in the discussion. It could probably well be their effect estimates and subsequent p-values are suffering from the “winner`s curse”. The independent cohort validation is a must before any meaningful inferences can be deduced from the present study.

Is the work clearly and accurately presented and does it cite the current literature?

Yes

If applicable, is the statistical analysis and its interpretation appropriate?

Yes

Are all the source data underlying the results available to ensure full reproducibility?

Yes

Is the study design appropriate and is the work technically sound?

Yes

Are the conclusions drawn adequately supported by the results?

Yes

Are sufficient details of methods and analysis provided to allow replication by others?

Yes

Reviewer Expertise:

Neurogenetics, Genetic Epidemiology, Statistical Genetics.

I confirm that I have read this submission and believe that I have an appropriate level of expertise to confirm that it is of an acceptable scientific standard.

# Peer review: Reviewer response for version 1

**DOI:** 10.21956/wellcomeopenres.19151.r47417

**Published:** 2021-12-14

**Authors:** Nicholas W. Wood

**Affiliations:** 1 Department of Clinical and Movement Neurosciences, UCL Queen Square Institute of Neurology, University College London, London, UK

An interesting and important study to evaluate the possible biologically motivated causal relationship between S100 and Alzheimer's disease. Too often biomarkers are reported for a variety of neurodegenerative diseases and no definite (or often plausible) biological link is made. Here, the authors have used 2-stage Mendelian randomisation to attempt to investigate whether SNPs associated with S100 levels and/or a methylation epigenetic readout are causally related to Alzheimer's disease.

 They first performed an analysis in the Lothian birth cohort to 'build' the eugenic instruments and then applied them to the very large GWAS datasets available for Alzheimer's. They do not find any evidence that genetically encoded determination of S100 and its methylation is linked to AD. This is useful to the community.

 My minor comments:

- Could they detail what amount of the variance of expression is captured by the instrument?
- Also, I think for clarity, explaining a bit more about the trio probability assessments in the Coloc analyses would help the non-specialist reader.

Is the work clearly and accurately presented and does it cite the current literature?

Yes

If applicable, is the statistical analysis and its interpretation appropriate?

Yes

Are all the source data underlying the results available to ensure full reproducibility?

Partly

Is the study design appropriate and is the work technically sound?

Yes

Are the conclusions drawn adequately supported by the results?

Yes

Are sufficient details of methods and analysis provided to allow replication by others?

Yes

Reviewer Expertise:

Neurogenetics

I confirm that I have read this submission and believe that I have an appropriate level of expertise to confirm that it is of an acceptable scientific standard.
